# Analysis of Functional Genomic Signals Using the XOR Gate

**DOI:** 10.1371/journal.pone.0005608

**Published:** 2009-05-19

**Authors:** Mahesh Yaragatti, Qi Wen

**Affiliations:** 1 Biotechnology Program, University of Pennsylvania, Philadelphia, Pennsylvania, United States of America; 2 Department of Physics, University of Pennsylvania, Philadelphia, Pennsylvania, United States of America; Tel Aviv University, Israel

## Abstract

Modeling gene regulatory networks requires recognition of active transcriptional sites in the genome. For this reason, we present a novel approach for inferring active transcriptional regulatory modules in a genome using an established systems model of bit encoded DNA sequences. Our analysis showed variations in several properties between random and functional sequences. Cross correlation within random and functional groups uncovered a wave pattern associated with functional sequences. Using the exclusive-OR (XOR) logic gate, we formulated a scheme to threshold signals that may correlate to putative active transcriptional modules from a population of random genomic fragments. It is our intent to use this as a basis for identifying novel regulatory sites in the genome.

## Introduction

A digital transformation of genomic sequences has facilitated the biological discovery of regulatory regions in the genome through computational methods. Since 2003, the ENCODE Consortium has endeavored to identify all the regulatory sites in the human genome; however, there are still 10^5^ more regulatory sites than genes in the mammalian genome. This requires the development of creative approaches to identify novel transcriptional regulatory sites in the genome.

There is opulent literature suggesting that one of the primary difficulties in locating active transcriptional sites is due to the majority of the mammalian genome being transcribed and most regulatory sequences being multi-functional [1], [2], [3], [4]. Although we are not presenting results to challenge this idea, it is implicit in our approach that our randomly selected fragments or mini-modules (150–250 bp) from the genome are more likely non-functional by comparison to the experimentally validated active sequences.

It is arguable that our method may only be detecting compositional differences between sequences which may not correlate to functionality. However, regulatory sequences represent a broad array of components including enhancers, repressors, silencers, and other boundary elements. Hence, the genomic data presently available to define specific properties of these individual components is very non-specific since most sites have not yet been discovered in the mammalian genome [5].

Digital delineation of genomic sequences such as DNA sequence barcoding [6] and implementation of boolean logic gates have been widely employed to study transcription [7], [8], [9]. Unfortunately, there has been limited advancement on the application of boolean logic gates and regulatory module bit coding. In this paper, we used an established systems model of bit encoded sequences from Yaragatti et al. (2009) to identify potentially active modules in the genome through a signal plot. The rationale for our approach was to evaluate the interaction of discrete properties between sequences.

## Methods

### Genomic Sequence Database

The random and functional genomic sequences analyzed in this paper were acquired from Yaragatti et al. (2009). As reported, the functional sequences were experimentally validated as enhancers in a mammalian F9 cell line to increase the level of GFP expression when inserted upstream of a promoter site. The average size of these fragments was 149 bp. The random sequences had lengths >150 and <250 and were selected from the mouse genome (UCSC mm9, July 2007 Assembly). As reported, these sequences were encoded as bit vectors based on genomic features (from the UCSC Genome Browser) that were either present (equals 1) or not present (equals 0) in the sequences. These sequences were not modified in any way from their original source and additional details can be found in their online *Bioinformatics* Supplemental Table 2.

### Cross Correlation and XOR Implementation

We implemented the following functions using Matlab scripts to compare a genomic sequence (vector) to other sequences in our dataset. The cross correlation (xcorr) function is a standard built in function that computes raw correlations with no normalization. The xcorr(x,y) function returns the cross-correlation sequence in a length 2*N−1 vector, where x and y are vectors of length *N* (*N*>1).
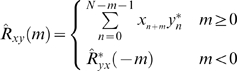
The output vector c has elements given by *c*(*m*) = *R*_xy_(*m*−*N*), *m* = 1, …, 2*N*−1.

A very similar function to xcorr is the exlusive-OR (xor) operation. We used xor(x, y) to perform an operation on the corresponding elements of vectors x and y. The resulting element C(i,j,…) is logically true (i.e., equal to1) if x(i,j,…) or y(i,j,…)–but not both–is nonzero. In both applications, we summed the resultant vector and plotted the normalized scores for each vector.

Averaging the XOR scores for the functional sequences provides a threshold value for isolating putative functional sequences from the random genomic sequence population. All Matlab scripts used in our analysis are available in Figure S2.

### Statistical Analysis

Chi square analysis was performed through a built-in function (with default settings) using Stata v.9E software. All data reported has signficance at the 95% confidence level (i.e., p<0.05). The reported t-values were acquired from the online *Bioinformatics* Supplemental Table 1 and were included in our table for comparison purposes.

## Results and Discussion

This paper introduces a novel scheme to isolate candidate functional mini-modules from a random population of genomic fragments based on sequence properties. In an earlier study, it was successfully shown that our genomic fragments can be classified into active and inactive groups by calculating a composite score for each sequence and analyzing it through a support vector machine [10]. We are expanding on this analysis by creating a model that evaluates the interaction of these genomic vectors using the same set of sequence features. In our initial analysis, we presented a t-test that compared the means between vectors to show variation between the groups. To support our current model, a chi square (χ^2^) test was performed on the features, by comparing frequency, to justify differences between elements of the vectors. We observed a wider range of χ^2^ values, but the results from the chi square analysis are essentially similar to the t-test (Figure S1).

**Figure 1:**
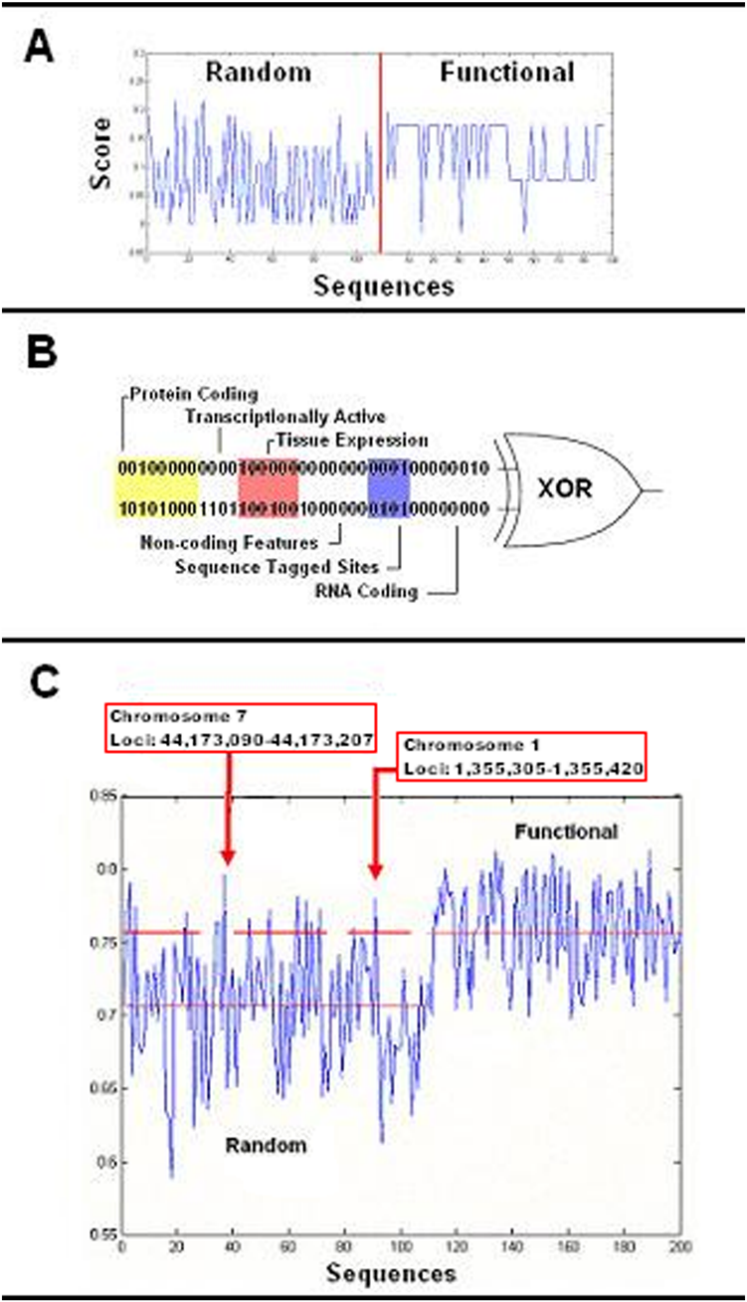
Analysis of bit encoded genomic sequences. (a) Each sequence (point) is evaluated against every other sequence outputting a binary vector which is summed and normalized. The correlation coefficient was performed on random and functional vectors (t = 6.19, f = 15.17, df = 201, p<0.05). The correlation coefficient is a number between 0 and 1 and if there is no relationship between the compared sequences, then a value of 0 is predicted. (b) Schematic of XOR model used for bit encoded genomic sequences. (c) By using the XOR function, we can select sequences from the random population based on a threshold value from the functional modules (t = 5.52, f = 4.91, df = 201, p<0.05).

We computed the correlation coefficient to show variation between random and functional sequences with the assumption that the absolute score of a sequence does not directly relate to its functionality. As shown in Figure 1A, the random sequences generated a large range of scores whereas the functional sequences were limited to approximately three values suggesting there was limited variation within the functional group. The rationale for the XOR logic gate (Figure 1B) was to emulate the binary output function of the correlation coefficient with the major exception that similar sequences (i.e., vectors having similar elements) would score equivalently. We implemented a scheme in Figure 1C whereby random sequences with higher scores are more likely to be functional transcriptional modules, which can be selected by calculating thresholds. Although reported in our earlier study, it is important to bear in mind that the random sequences may have contained genic, intergenic, and non-coding regions of the genome.

An important aspect of the support vector machine approach used in Yaragatti et al. (2009) was that each sequence was evaluated by the integration of its discrete features. This paper proposes an analogous model that relates a sequence to other sequences (i.e., the interaction of features) for predicting functionality. This approach may uncover modules active in a specific regulatory network (i.e., transcriptional regulation of a gene) that may otherwise go undetected using current computational methods.

Using the mathematical XOR function, we have introduced a novel approach to approximate loci-specific transcriptional active sites in the genome. This is a simple, but effective, approach that interprets functional DNA as a dynamic bit code of features rather than a static sequence of nucleotide based motifs. Ultimately, we intend to advance this paradigm of analyzing regulatory sequences using bit vectors in more complicated systems with the hope of elucidating the functional landscape of eukaryotic genomes.

## Supporting Information

Figure S1 Chi square analysis was performed on features that were used in our cross correlation and XOR logic gate.
(0.85 MB TIF)

Figure S2 Matlab Scripts for Implementation of Cross Correlation and XOR Logic Gate.
(0.04 MB DOC)
