# Supplementary figures and images for: Analysis of Functional Genomic Signals Using the XOR Gate
Source: PLoS One. 2009 May 19;4(5):e5608. doi: 10.1371/journal.pone.0005608 (PMC2680033; doi:10.1371/journal.pone.0005608)

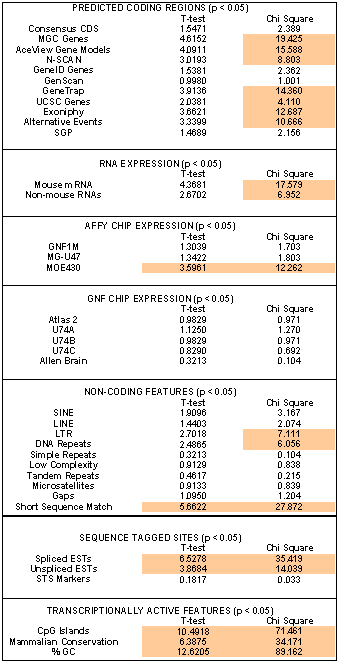

Supplement: Figure S1 — Chi square analysis was performed on features that were used in our cross correlation and XOR logic gate. (0.85 MB TIF) [file pone.0005608.s001.tif]
