# Supplementary material for: Analysis of Functional Genomic Signals Using the XOR Gate
Source: PLoS One. 2009 May 19;4(5):e5608. doi: 10.1371/journal.pone.0005608 (PMC2680033; doi:10.1371/journal.pone.0005608)
Supplement: Figure S2 — Matlab Scripts for Implementation of Cross Correlation and XOR Logic Gate. (0.04 MB DOC) [file pone.0005608.s002.doc]

**FIGURE S2**

| **XOR Logic Gate (MATLAB)** |
| --- |
| clear all  datad=xlsread('set.xls');  realv=datad(110:200,:);  veclen=size(datad,2);  for i=1:size(datad,1)  vc(i)=0;  for j=1:size(realv,1);  corrs=xor(realv(j,:),datad(i,:));  scor=sum(1-corrs)/veclen;  vc(i)=vc(i)+scor;  end  vc(i)=vc(i)/j;  end    binlocs=[0:0.02:1];  figure(1)  plot(vc)  hold on  plot([1:109],mean(vc(1:109))*ones(1,109),'r')  plot([110:200],mean(vc(110:200))*ones(1,91),'r')  ylabel('Score');  xlabel('vector #');  title('Score of vectors')  hold off  xlim([0 310])  ylim([0.55 0.85]);    %title('Distribution of score for other vectors');  n1=hist(vc(1:109),binlocs)/109;  n2=hist(vc(110:200),binlocs)/91;  figure(2)  plot(binlocs,n1,'--or','MarkerFaceColor','r','MarkerSize',9)  hold on  plot(binlocs,n2,'--ob','MarkerFaceColor','b','MarkerSize',9)  hold off  xlim([0.45 0.95]);  legend('random vector', 'real vector') |

| **Cross Correlation (MATLAB)** |
| --- |
| randv=datad(1:109,:);  realv=datad(110:200,:);  for i=1:size(randv,1)  cor=xcorr(randv(i,:),randv(2,:));  corrc(i)=cor(size(randv,2)-1)/size(randv,2);  end  plot(corrc)  for i=1:size(realv,1)  cor=xcorr(realv(i,:),realv(2,:));  corrcv(i)=cor(size(realv,2)-1)/size(realv,2);  end  figure,plot(corrcv)  for i=1:size(othev,1)  cor=xcorr(othev(i,:),othev(2,:));  corrov(i)=cor(size(othev,2)-1)/size(othev,2);  end  figure,plot(corrov) |
